# The Effect of Intra-abdominal Pressure on Heart Rate Variability and Hemodynamics During Laparoscopic Cholecystectomy: A Prospective Observational Study

**DOI:** 10.7759/cureus.57890

**Published:** 2024-04-09

**Authors:** Nurhan Eren, Didem Onk, Alparslan Koç, Ufuk Kuyrukluyildiz, Remziye Ayşenur Nalbant

**Affiliations:** 1 Department of Anesthesiology, Erzincan Binali Yıldırım University Mengucek Gazi Training and Research Hospital, Erzincan, TUR; 2 Department of Anesthesiology and Reanimation, Erzincan Binali Yıldırım University, Erzincan, TUR

**Keywords:** cardiac autonomic neuropathy, heart rate variability, intraabdominal pressure, laparoscopic cholecystectomy, diabetes mellitus

## Abstract

Introduction

This study aimed to evaluate hemodynamic changes using heart rate variability (HRV) measurements in diabetic and nondiabetic patients who will undergo laparoscopic cholecystectomy and to provide our preoperative measurements to guide us for better perioperative anesthesia management.

Materials and methods

The study included 143 patients aged 40 years and older who would undergo elective laparoscopic surgery, did not have any comorbidities other than diabetes mellitus (DM) type II, and were in the American Society of Anesthesiologists (ASA) class I-III risk group. Patients were divided into two groups: the control group (n = 77) and the DM group (n = 66). The preoperative glycated hemoglobin (HbA1C) level was measured. Peripheral oxygen saturation (SpO2) and hemodynamic parameters such as systolic arterial pressure (SAP), diastolic arterial pressure (DAP), mean arterial pressure (MAP), heart rate (HR), and HRV parameters were measured preoperatively, perioperatively, and postoperatively. Intra-abdominal pressure (IAP) was administered at 10-12 mmHg.

Results

Even though SAP, DAP, MAP, and HR decreased with induction, they increased with insufflation, and an overall decrease was seen at the postoperative 24th hour for all parameters. When the groups were evaluated, no difference was observed except that the DAP was significantly lower in the DM group (p = 0.029) at insufflation and the HR was higher in the DM group at induction, and the difference was significant (p = 0.001). Preoperative HRV parameters were significantly lower in the DM group. According to the HRV parameters, although a decrease was observed after induction and insufflation, conversely, an increase was observed postoperatively. When the postoperative and preoperative values were compared, the standard deviation of the NN (R-R) intervals (SDNN), SDNN index, high frequency (HF), low frequency (LF), and LF/HF parameters were found to be significantly lower in the DM group than in the control group.

Conclusion

Diabetic patients are more sensitive to increased intra-abdominal pressure (IAP) in laparoscopic surgery, and the effects on cardiac autonomic functions can be determined by HRV measurements without clinically reflecting on hemodynamic data. Additionally, in diabetic patients with preoperative LF and/or HF values less than 100, we believe that careful follow-up in terms of autonomic neuropathy complications and anesthesia management should be done more meticulously in these patients.

## Introduction

Diabetes and the onset of related complications are considered serious health problems worldwide. While the most substantial cause of mortality in diabetes is cardiovascular disease, diabetic autonomic neuropathy is also a frequent and serious complication. The incidence of autonomic neuropathy among diabetic patients at one year, five years, and 20 years is 4%, 20-30%, and 30-50%, respectively [1]. Several factors can affect the cardiovascular system response in patients who undergo laparoscopic surgery, including the intra-abdominal pressure (IAP), the position of the patient, and CO2 absorption. An increase of more than 10 mmHg in abdominal pressure could result in significant hemodynamic changes [2,3]. Such an increase causes an elevation in the intrathoracic pressure and vascular resistance, leading to elevated arterial pressure and a decreased flow rate of the heart [4]. Meanwhile, the heart rate may not be affected or can be slightly elevated [5]. Whether in the prone or supine position, an approximately 10-30% decrease in cardiac flow has been demonstrated during the peritoneal insufflation [6,7]. Although cardiac flow decreases with peritoneal insufflation, it increases reciprocally with surgical stress [8].

Heart rate variability (HRV) is defined as the standard deviation between two consecutive R waves in an electrocardiogram (ECG) signal. Since it demonstrates the precise impact of both sympathetic and parasympathetic nerves during measurement, HRV may also be defined as cyclic changes in the sinus rhythm. Therefore, it is considered an indicator of both the cardiorespiratory system and cardiac autonomic tone [9,10]. Since any dysfunction in the autonomic nervous system could complicate peroperative conditions in surgical patients, we believe that routine preassessment of the autonomic nervous system is essential for daily anesthesia practice.

This study aimed to investigate the changes in hemodynamics and HRV parameters in patients due to intra-abdominal pressure (IAP) following laparoscopic cholecystectomy. And also, aimed to use the preoperative assessment results for the guidance of our peroperative anesthesia management. For this purpose, we examined the hemodynamic responses of diabetic patients with unknown autonomic neuropathy to increased IAP during laparoscopic surgery.

## Materials and methods

The study was conducted at Erzincan Binali Yıldırım University Mengucek Gazi Training and Research Hospital, Erzincan, Turkey. After obtaining permission from the ethics committee of Erzincan Binali Yıldırım University (Date: 26.03.2019 No: 02/10), a total of 143 patients who were planned to go under elective laparoscopic cholecystectomy over 40 years of age with American Society of Anaesthesiologists (ASA) physical status classification I-III were included in the prospective observational study and obtained individual written consents. For the sample size calculation, the study conducted by Colombo et. al. was used [11]. According to the study, there was a 79% increase between the preoperative and induction times. Based upon the hypothesis that the same changes should be seen with the same carbon dioxide pressures, the sample size was calculated to be 130, with α values of 0.05 and 80% power.

Patients with the following conditions were excluded from the study: age less than 40 years; diagnosed with type I diabetes mellitus (DM); diagnosed with uncontrolled hypertension and/or arrhythmias; exhibiting alcohol and/or drug addiction; classified as ASA risk class IV-V; presenting with comorbidities that can increase abdominal pressure and/or cause pulmonary hypertension; undergoing laparoscopy but needed to be switched to laparotomy during the procedure; experiencing shock; decompensated heart or pulmonary failure; kidney or liver failure; with peripheral neuropathy and/or chronic neurologic/cardiovascular diseases; documented allergies to the anesthesia medications; diagnosed with obesity or requiring regional anesthesia.

The past medical records of each patient were evaluated for confirmation of the DM diagnosis. Patients were separated into two groups: type II DM (+) and type II DM (-), according to their comorbidities.

The following measurements were obtained and recorded for every patient: fasting blood sugar; glycated hemoglobin (HbA1c); oxygen saturation (SpO2); hemodynamic parameters such as systolic arterial pressure (SAP); diastolic arterial pressure (DAP); mean arterial pressure (MAP) and heart rate (HR); HRV parameters such as standard deviation of the NN (R-R) intervals (SDNN); SDNN index; the proportion of NN50 divided by the total number of NN intervals (PNN50); root mean square of the successive differences (rMSSD); triangular index; high frequency (HF); low frequency (LF); LF/HF; intraoperative and postoperative SpO2.

All participants were premedicated with midazolam (0.02 mg/kg), and premedicated patients were taken to the operating room and monitored (electrocardiogram (ECG), blood pressure, heart rate, peripheral oxygen saturation, HRV) as a routine. Operations took place under general anesthesia as always. The intra-abdominal pressure was set at 10-12 mmHg for all laparoscopic cholecystectomy patients by the same surgical team [12-17].

Hemodynamic HRV parameters were measured in the following period: T0: preoperative; T1: after induction; T2: after pneumoperitoneum (PP); T3: postoperative 24 hours.

Heart rate variability parameters were obtained as frequency- and time-dependent variables via the mini-holter recorder BI 9800 TL+3 (Biomedical Instruments Co.), and the calculations were done using the Ambulatory ECG (ECGLAB) system program. Hemodynamic and HRV values were compared between groups subsequently.

Statistical analysis

While the continuous variables were defined via mean ±, standard deviation (SD), median, minimum, and maximum values, the categorical variables were defined via n (%) in terms of descriptive statistics. A chi-square test was used for the analysis of categorical variables like sex and change ratio (e.g., above or below 50%). A Fisher exact test was used in cells with lower-than-expected values. Shapiro-Wilk’s test was used to confirm the normal distribution of the continuous variables. The percent alteration of age and HRV parameters (%ΔT0-T3) between groups was compared with the Mann-Witney U test. The fluctuation of HRV parameters’ over time was analyzed via a repeated measures analysis of variance (ANOVA) test. Mauchly’s test was used for testing the assumption of sphericity, and the Greenhouse-Geisser test was used if the assumption was not met. A Bonferroni multiple comparison-correction test was used to determine which groups differed. Spearman correlation analysis was used for the relation between continuous variables. A p-value of <0.05 was considered statistically significant. The data analysis was done using IBM SPSS Statistics for Windows, Version 22 (released 2013; IBM Corp., Armonk, New York, United States).

## Results

A total of 143 patients were included in the study, of which 77 were (53.8%) in the control group and 66 were (46.2%) in the DM group. There were 98 women (68.5%) and 45 men (31.5%) in total. While the age average was 54.8±10.6 (min:40, max:84) in the control group, it was 57.6±10.1 (min:40, max:78) in the DM group, and there was no significant difference (p = 0.091) between groups. Female dominance was seen in both groups, with no significant difference (p = 0.317) in between (Table 1).

**Table 1:** Baseline characteristics of the study population Data in the table are presented using means and standard deviations (mean ± SD) for continuous variables and counts with percentages n (%) for categorical variables. ^*^p <0.05 is considered significant; DM: diabetes mellitus

		Control	DM	p-value
Age, (Mean±SD)		54.8±10.6	57.6±10.1	0.091
Sex	Male	27 (35.1)	18 (27.3)	0.317
	Female	50 (64.9)	48 (72.7)	

When the overall averages between groups were analyzed for time periods (T0, T1, T2, and T3), there was no significant difference for the SAP (p = 0.857), DAP (p = 0.245), MAP (p = 0.650), and HR (p = 0.088) values. While there was no difference for the SAP and MAP values in groups overall, the DAP was higher (p = 0.029) after pneumoperitoneum (T2) and the HR was lower (p = 0.001) after induction (T1) in the diabetic group (Table 2).

**Table 2:** Time-dependent variation of the heart rate and arterial pressure SAP: systolic arterial pressure; DAP: diastolic arterial pressure; MAP: mean arterial pressure; HR: heart rate; DM: diabetes mellitus The data in the table are presented as means and standard deviations (mean ± SD) and median (median, min-max) values for continuous variables. *Variance analysis was used for repeated measurements. Bonferonni correction was used for group comparison at time periods. p <0.05 is considered significant.

	Control		DM		p-value
	Mean±SD	Median(min-max)	Mean±SD	Median(min-max)	
SAP-T0	127.0±12.5	128.0(98.0-160.0)	128.8±19.7	128.5(78.0-168.0)	0.504
SAP-T1	121.0±12.2	120.0(90.0-162.0)	118.9±18.1	117.5(76.0-156.0)	0.412
SAP-T2	126.2±13.9	126.0(86.0-161.0)	131.3±21.0	131.5(80.0-174.0)	0.082
SAP-T3	119.4±10.7	120.0(99.0-140.0)	116.3±17.2	116.0(77.0-154.0)	0.194
DAP-T0	79.2±10.2	80.0(53.0-104.0)	81.6±14.0	81.5(49.0-108.0)	0.251
DAP-T1	75.3±10.7	76.0(50.0-104.0)	76.4±11.9	77.0(50.0-103.0)	0.573
DAP-T2	79.1±10.8	79.0(55.0-104.0)	83.7±14.4	83.5(50.0-114.0)	0.029^*^
DAP-T3	74.0±10.2	74.0(50.0-96.0)	74.6±11.9	74.5(48.0-100.0)	0.753
MAP-T0	111.1±11.0	113.0(84.0-134.0)	113.0±17.5	113.0(68.0-148.0)	0.426
MAP-T1	105.7±11.2	105.0(79.0-143.0)	104.8±15.9	104.0(67.0-138.0)	0.663
MAP-T2	110.5±12.2	111.0(77.0-142.0)	115.5±18.7	115.5(70.0-154.0)	0.059
MAP-T3	104.3±10.1	104.0(83.0-125.0)	102.5±15.3	102.0(67.0-134.0)	0.391
HR-T0	83.7±11.9	83.0(57.0-124.0)	79.3±16.8	79.0(43.0-117.0)	0.069
HR-T1	80.1±14.3	80.0(55.0-120.0)	72.3±13.9	70.5(50.0-118.0)	0.001^*^
HR-T2	81.2±10.9	79.0(58.0-114.0)	83.2±16.4	81.0(49.0-125.0)	0.376
HR-T3	78.7±14.5	79.0(55.0-112.0)	76.0±15.2	73.5(49.0-121.0)	0.277

In the analysis of the overall averages between groups, SDNN (p <0.001), SDNN index (p <0.001), rMSSD (p = 0.003), and PNN50 (p = 0.024) parameters were found to be lower in the DM group. While the SDNN and SDNN index parameters were lower at all time periods, rMSSD was lower at insufflation (T2) and postoperative (T3) periods, and PNN50 was lower at preoperative (T0) and insufflation (T2) periods in the DM group (Table 3).

**Table 3:** Time-dependent variation of time-dependent HRV parameters SDNN: standard deviation of the NN (R-R) intervals; SDNN index: standard deviation of the averages of NN intervals for all five min segments; rMSSD: root mean square of the successive differences; PNN50: the proportion of NN50 divided by the total number of NN intervals; HRV: heart rate variability; DM: diabetes mellitus The data in the table are presented as means and standard deviations (mean ± SD) and median (median, min-max) values for continuous variables. *Variance analysis was used for repeated measurements. Bonferonni correction was used for group comparison at time periods. p <0.05 is considered significant.

	Control		DM		p-value
	Mean±SD	Median(min-max)	Mean±SD	Median(min-max)	
SDNN-T0	55.7±15.8	52.0(27.0-121.0)	49.3±14.0	48.0(22.0-88.0)	0.013^*^
SDNN-T1	38.9±13.0	37.0(19.0-92.0)	28.9±9.7	28.0(12.0-56.0)	<0.001^*^
SDNN-T2	33.1±12.1	31.0(14.0-83.0)	24.9±9.0	24.5(10.0-49.0)	<0.001^*^
SDNN-T3	50.0±15.8	47.0(25.0-115.0)	36.3±14.2	33.5(14.0-78.0)	<0.001^*^
SDNN Index-T0	46.1±12.6	45.0(21.0-90.0)	35.5±11.6	34.0(14.0-66.0)	<0.001^*^
SDNN Index-T1	25.7±8.8	24.0(12.0-52.0)	19.7±8.6	18.5(7.0-42.0)	<0.001^*^
SDNN Index-T2	23.7±8.8	22.0(10.0-54.0)	18.4±8.0	18.0(6.0-40.0)	<0.001^*^
SDNN Index-T3	44.5±13.0	44.0(20.0-88.0)	29.6±12.9	27.0(11.0-68.0)	<0.001^*^
rMSSD-T0	27.9±13.4	27.0(8.0-89.0)	24.0±10.8	23.0(7.0-52.0)	0.054
rMSSD-T1	24.5±16.5	20.0(7.0-99.0)	20.2±8.9	17.0(10.0-50.0)	0.060
rMSSD-T2	24.0±12.1	21.0(7.0-70.0)	19.8±10.7	16.0(9.0-56.0)	0.030^*^
rMSSD-T3	27.4±10.6	27.0(9.0-59.0)	21.3±9.6	19.0(10.0-59.0)	<0.001^*^
PNN50-T0	8.7±8.5	7.0(0.0-46.0)	6.2±6.2	5.0(0.0-29.0)	0.049^*^
PNN50-T1	6.7±7.9	3.0(0.0-46.0)	5.2±8.1	2.0(0.0-40.0)	0.257
PNN50-T2	6.6±9.2	3.0(0.0-46.0)	4.0±5.6	2.0(0.0-28.0)	0.049^*^
PNN50-T3	9.8±8.8	7.0(0.0-37.0)	6.8±9.3	3.0(0.0-44.0)	0.053

The LF and LF/HF parameters were found to be lower in all time periods in the DM group. While the HF values were similar at the preoperative (T0) measures and remained in this trend after induction (T1) and insufflation (T2), they were found to be lower at the postoperative 24th hour (T3) in the DM group (Table 4).

**Table 4:** Time-dependent variation of frequency-dependent HRV parameters LF: low frequency;  HF: high frequency; LF/HF: LF to HF ratio The data in the table are presented as means and standard deviations (mean ± SD) and median (median, min-max) values for continuous variables. *Variance analysis was used for repeated measurements. Bonferonni correction was used for group comparison at time periods. p <0.05 is considered significant.

	Control		DM		p-value
	Mean±SD	Median(min-max)	Mean±SD	Median(min-max)	
HF-T0	210.6±170.1	157.9(44.5-887.5)	178.6±154.7	111.9(38.1-693.4)	0.244
HF-T1	107.0±81.9	84.5(14.7-368.7)	103.8±117.7	56.8(10.9-508.4)	0.849
HF-T2	86.7±70.1	61.7(2.6-318.5)	73.3±93.6	32.5(6.0-436.5)	0.333
HF-T3	172.7±143.8	133.6(22.2-875.1)	120.2±117.4	70.8(18.0-539.3)	0.019^*^
LF-T0	325.7±215.7	265.7(45.0-873.2)	178.6±117.7	139.0(40.0-453.7)	<0.001^*^
LF-T1	147.1±95.9	137.0(13.2-402.1)	78.9±60.1	56.8(15.8-279.8)	<0.001^*^
LF-T2	112.3±75.8	109.2(2.1-282.5)	56.5±58.1	30.1(1.7-212.2)	<0.001^*^
LF-T3	266.8±189.5	236.4(14.0-894.8)	99.9±78.3	67.8(18.9-343.7)	<0.001^*^
LF/HF-T0	1.74±0.78	1.6(0.4-5.5)	1.22±0.67	1.0(0.5-2.7)	<0.001^*^
LF/HF-T1	1.69±1.90	1.4(0.2-16.4)	1.15±0.97	0.8(0.1-3.7)	0.040^*^
LF/HF-T2	1.62±0.97	1.5(0.2-7.7)	1.0±0.59	0.8(0.2-2.4)	<0.001^*^
LF/HF-T3	1.78±1.06	1.6(0.2-7.4)	1.04±0.47	1.1(0.2-1.9)	<0.001^*^

When the correlation between HbA1c and HRV was analyzed, no correlation was found with the triangular index (triangle), yet a negative correlation was found with all the remaining parameters (Table 5).

**Table 5:** Correlation of HbA1c with HRV parameters SDNN: standard deviation of the NN (R-R) intervals; SDNN index: standard deviation of the averages of NN intervals for all five min segments; rMSSD: root mean square of the successive differences; PNN50: the proportion of NN50 divided by the total number of NN intervals; LF: low frequency; HF: high frequency; LF/HF: ratio of LF to HF *r: Correlation factor, if the correlation coefficient is between r = 0-0.39, it indicates a weak correlation; if it is between r = 0.40-0.69, it indicates a moderate correlation; and if it is r = 0.70 and above, it indicates a high correlation. **p <0.05 is considered significant.

		SDNN	SDNN İndex	rMSSD	pNN50	Triangle	HF	LF	LF/HF
HbA1c	r*	-0.267	-0.432	-0.176	-0.186	-0.034	-0.249	-0.434	-0.381
	p	0.001^**^	<0.001^**^	0.036^**^	0.026^**^	0.689	0.003^**^	<0.001^**^	<0.001^**^

In both groups, the postoperative values of SDNN, SDNN index, HF, LF, and LF/HF were found to be lower than the preoperative values. This decrease was found to be more profound in the DM group (Table 6).

**Table 6:** Percentage changes of postoperative HRV values compared with the preoperative values SDNN: standard deviation of the NN (R-R) intervals; SDNN index: standard deviation of the averages of NN intervals for all five min segments; rMSSD: root mean square of the successive differences; PNN50: the proportion of NN50 divided by the total number of NN intervals; LF: low frequency; HF: high frequency; LF/HF: ratio of LF to HF; DM: diabetes mellitus *The Mann-Whitney U test was used. ** The preoperative and postoperative LF/HF change in the DM group was not significant (p = 0.059). Yet the change in percentage was significantly higher than the control group(p = 0.040), p <0.05 is considered significant.

%	Control		DM		p-value
	Mean±SD	Median	Mean±SD	Median	
SDNN	10.7±6.3	10.2	27.5±12.7	28.4	<0.001^*^
SDNN index	3.4±11.2	4.6	18.4±14	19.6	<0.001^*^
rMSSD	17±71.7	3.7	0.3±43.3	6.3	0.259
PNN50	150.1±479.9	0	26±164.9	0	0.057
Triangle	16.3±89.6	28.5	27.6±34.6	36.3	0.646
HF	17.7±23.1	15.2	35.1±17.9	31.9	<0.001^*^
LF	18.5±22.9	15.4	41.9±24.9	40.4	<0.001^*^
LF/HF**	1.2±25.3	0.2	6.5±40	10.8	0.040^*^

A higher than 50% postoperative depression ratio was seen more frequently if the preoperative HF and/or LF value was measured below 100 compared with the contrary of above 100 [(p = 0.005(K), p <0.001(DM)].

In subjects with preoperative HF and/or LF below 100, there was a significant difference between the DM and control groups in terms of depression ratios (77.1% and 38.1, respectively) (p = 0.003).

In subjects with postoperative HF and/or LF above 100, there was a significant difference between the DM and control groups in terms of depression ratios (29.0% and 8.9, respectively) (p = 0.03) (Table 7).

**Table 7:** Suppression rates of postoperative HF and LF compared with the preoperative values LF: low frequency; HF: high frequency; LF/HF: ratio of LF to HF; DM: diabetes mellitus The absence of numerical data instead indicates the presence of p-values. *The Fisher Exact test was used for the analysis of percentage changes.** P-value for the comparison between groups. p <0.05 is considered significant.

Group				HF and/or LF <100	HF and LF >100	Total	p-value
Control	HF LF change (%ΔT0-T3)	<50%	n	13	51	64	0.005^*^
			%	61.9%	91.1%	83.1%	
		>50%	n	8	5	13	
			%	38.1%	8.9%	16.9%	
DM	HF LF change (%ΔT0-T3)	<50%	n	8	22	30	<0.001^*^
			%	22.9%	71.0%	45.5%	
		>50%	n	27	9	36	
			%	77.1%	29.0%	54.5%	
p**	-	-	-	0.003^*^	0.030^*^	-	-

## Discussion

Diabetes-related neuropathy combined with a heart problem is a significant long-term consequence. Hemodynamic instability during induction and trouble-sustaining anesthesia may result from this [18]. We found the diabetic group to be more sensitive to the increasing intra-abdominal pressure, as well as having lower HRV parameters after laparoscopic surgery. When the patients with preoperative HF and/or LF parameters lower than 100 were analyzed, we found that postoperative HRV values decreased more specifically in diabetic patients. The age distribution between groups was similar, with the diabetic group’s average being 57.6 and the control group's being 54.8. As the most contributing factor to HRV is age, this similarity allows our study not to be affected by hemodynamic factors that may develop due to age. While the fasting blood sugar and HbA1C parameters were found to be significantly higher, the HRV measurements were found to be lower in the DM group. Even though the current literature shows that HbA1C is the most contributing factor to the frequency-dependent HRV parameters, there’s no agreement about which parameter is the earlier determinant factor [19].

Evaluation of hemodynamic parameters between groups shows that DAP has increased more after insufflation and HR has decreased more after induction in the DM group. We think that the increase in the DAP and the decrease in the HR are caused by the DM group being more sensitive to the increasing IAP and given anesthetic medications, respectively. We believe that the similar results of the hemodynamic data at other measurement times are due to patients not having diabetes-related complications and all surgeries being performed by the same general surgeon. In preoperative measurements, SDNN, SDNN index, PNN50, LF, and LF/HF values were found to be significantly lower in the diabetic group. The meta-analysis results of Benichou et al. are in line with our findings; however, HF values were found to be lower in the diabetic group than in the control group, and no difference was found in LF/HF. We believe that Benichou et al.'s use of 24-hour recordings with a much larger study group (2932 patients) played a role in the different results [20]. While SDNN, SDNN index, triangular index, HF, and LF parameters decreased in both groups after induction, SDNN, SDNN index, and LF values were significantly lower in the diabetic group. In the literature, we could not find data on which parameters were early determinators in diabetic patients. In our study, SDNN, SDNN index, and LF values were found to be significantly lower, suggesting that these parameters may shed light on more comprehensive studies afterward. While a decrease in SDNN, SDNN index, HF, and LF parameters were found after insufflation, conversely, an increase in triangular index was detected in both groups. When we compared the LF/HF value measured after insufflation with the preoperative value, even though no significant difference was found in the control group, a significant decrease was seen in the diabetic group (p = 0.038). The fact that the LF/HF ratio decreased from the third minute of PP in the study conducted by Iorio et al. in 2010 is consistent with our diabetic group; this finding suggests that the diabetic group is more sensitive to increased intra-abdominal pressure [21].

When we examined our postoperative 24th-hour values, we found that SDNN, SDNN index, HF, and LF values increased in both groups while increasing more in the control group. Even though an increase was found in the control group in rMSSD and PNN50 parameters, there was no difference in the DM group. The reason for the increase in HRV parameters in our postoperative measurements is due to the disappearance of the effects of CO2 insufflation on the cardiovascular system and the recovery of cardiac autonomic functions over time [22]. While the hemodynamic data were similar between the groups, HRV parameters were found to be lower in the DM group. This finding suggests that HRV analysis can reveal diabetes-related complications before hemodynamic changes occur. The diagnosis and treatment of diabetic autonomic neuropathy by Ewing et al. also correlate with our studies’ results [23]. When we evaluated the changes in postoperative HRV values between the groups in comparison with the preoperative values, SDNN, SDNN index, HF, LF, and LF/HF parameters were significantly lower in the diabetic group than in the control group. In a different study, it was shown that the changes that decreased immediately after physical activity returned to normal after 24 hours; hence, cardiac recovery can be seen after 24 hours of such activities [24]. The fact that the diabetic groups’ results were lower in our study shows that increased intra-abdominal pressure affects this group more. HRV is the most commonly used and gold standard method for the diagnosis and follow-up of autonomic neuropathy. However, a single measurement does not suffice, and the changes from repeated measurements need to be evaluated [25]. When we evaluated our data, we found that the postoperative HRV values were suppressed for some of our subjects (LF and HF parameters), which was lower than 50% of their preoperative values. When we looked at the preoperative values of the patients whose postoperative values were suppressed, we found that the LF and/or HF values were mostly below 100. Postoperative suppression was also observed in patients with preoperative HF and LF values greater than 100. However, patients with HF and LF parameters less than 100 had significantly more postoperative suppression compared to patients with HF and LF parameters greater than 100 [p = 0.005 (C), p <0.001 (DM)]. In the diabetic group, we observed postoperative suppression to be significantly more frequent in patients with both preoperative LF and HF >100 (p = 0.030) and <100 measured (p = 0.003). We believe that this is due to lower than 100 HF and/or LF values and diabetes. Studies have shown that decreased LF and HF values carry a negative prognostic value for the cardiac autonomic neuropathy diagnosis [26].

Limitations

Even though our study aims to bring a new perspective to the literature, we couldn’t find the opportunity to compare our data because the HRV parameters differ among populations and do not have a predetermined cut-off value. With new comparative studies done in the future, it can be expected to determine cut-off values for the HRV parameters’ resulting in the usage of this parameter in clinical practice. We believe there’s a need for more comprehensive studies with larger sample sizes to do so.

## Conclusions

Low HRV values indicate low cardiac capacity. Autonomic nervous system dysfunction may complicate the problems that may happen preoperatively, thus aggravating the negative effects of the PP on DM patients. We believe that to protect the patients with HF and/or LF <100 from the negative hemodynamic effects that may be caused by PP, using lower pressure values (<10 mmHg), laparoscopy without gas, or other surgical methods such as open surgery would be useful and practical. Based on preoperative HRV measurements, we concluded that diabetic patients with HF and/or LF parameters less than 100 may be at risk for cardiac autonomic neuropathy. Also, we believe peroperative anesthetic management needs to be done more carefully with these patients.
